# Iatrogenic complete heart block due to His bundle transection in His lead placement

**DOI:** 10.1016/j.hroo.2024.12.013

**Published:** 2025-01-10

**Authors:** Bradley J. Reinoehl, Destino Roman, Joel Reinoehl

**Affiliations:** 1 Lake Erie College of Osteopathic Medicine, Erie, Pennsylvania; 2 Department of Electrophysiology, Bronson Advanced Cardiac Healthcare, Kalamazoo, Michigan

**Keywords:** Heart block, His bundle pacing, His-Purkinje system, Conduction system pacing, Ventricular pacing

**Key Findings**

▪ Conduction system pacing is emerging as the preferred method of long-term ventricular pacing.
▪ Conduction system pacing/lead placement is more complex than traditional right ventricular apical pacing.
▪ His bundle pacing carries a unique set of potential complications, which include damage to the conduction system.

## Introduction

His bundle pacing (HBP) is a widely used pacing technique to avoid the pitfalls of long-term right ventricular (RV) pacing. The recent increased use of HBP comes following evidence for a link between RV apical pacing and an increased risk for congestive heart failure (CHF). The concept of HBP is appealing due to the idea of physiologic pacing, which circumvents this risk of ventricular desynchrony and subsequent CHF. It has been long speculated that pacing the His bundle would produce a more physiologic ventricular activation. With new studies, the preservation of physiological pacing present in HBP has become evident.^1^ Due to technological limits, it was not until recently that HBP was investigated as an alternative for RV pacing.^2^ In several published reports, it was determined that HBP had potential use in permanent pacing.^3^^,^^4^ It would later be established that HBP has significantly improved outcomes in the treatment of chronic atrial fibrillation treated with pacemaker placement and atrioventricular (AV) nodal ablation.^5^ To preserve ventricular synchrony, HBP may be considered for atrial fibrillation patients being considered for the “pace and ablate” strategy.

Cardiac resynchronization therapy (CRT) is another potential area of use for HBP. Currently, the most used therapy for CRT was coronary sinus (CS) lead placement; however, it is not without its flaws.^6^ The implant failure rate for CS lead placement ranges from 5% to 9%.^7^ Additionally, rates of nonresponse to CS lead placement range between 30% and 40%.^8^ Due to the inconsistent nature of CS lead placement, it should come as no surprise that many alternatives to the procedure were studied. HBP is being investigated for the purpose of CRT; however, it is technically challenging.^9^ Left bundle branch pacing (LBBP) has been viewed as an alternative to biventricular pacing.^10^

One aspect of HBP that delayed its implementation was the lack of specialized procedural equipment and the technical difficulty of the procedure. HBP originally was performed with standard pacing leads, which were used to identify and pace the site of the largest His deflection detected. The increased indication for its use has led to significant development in equipment for HBP, with the development of a specialized pacing lead and sheath making the procedure much more efficient.^11^ The development of preshaped, steerable, and nonsteerable delivery sheaths, coupled with the widespread adoption of lumenless lead, has enabled precise delivery of lead in the His bundle.^12^

Several issues with HBP are well known: lead failure, high capture thresholds, prolonged procedure time, and early battery depletion. Lead failure is a rare but relevant risk when discussing HBP. Because of this risk, it has been common practice to place an RV lead as a backup to the His lead. Recent data suggest the prevalence of lead failure in HBP is less than originally thought, with Vijayaraman and colleagues reporting lead revision rate of 6.7% at 5 years. Another study described that in 100 patients that underwent HBP, 5 patients needed to have lead revisions.^13^ Capture threshold rise is another major issue with His leads. While no definitive pathology for rising capture threshold has been accepted, its predominance in short-term follow-up suggests an issue related to implantation technique.^14^ Beer and colleagues^14^ reported that approximately 15% of patients who undergo His lead placement experience a capture threshold increase of ≥1 V. Early battery depletion is another well-documented risk for HBP. The methodology of early battery depletion is due to other HBP complications. The current theory suggests that the RV backup lead and the higher capture threshold cause more rapid battery depletion. However, recent data suggest that most HBP patients do not have complications with premature battery depletion.^13^ Given the issues with HBP, LBBP has largely replaced HBP. When compared with HBP, LBBP has shown shorter procedure and fluoroscopy times and improved capture threshold and sensed R waves.^15^ The His bundle is located in the membranous septum. It continues into the muscular septum as the left bundle, where it divides into the anterior and posterior fascicles. The fibrous nature of the membranous septum may explain this threshold rise. We report a novel complication associated with HBP.

## Case presentation

**Figure 1:**
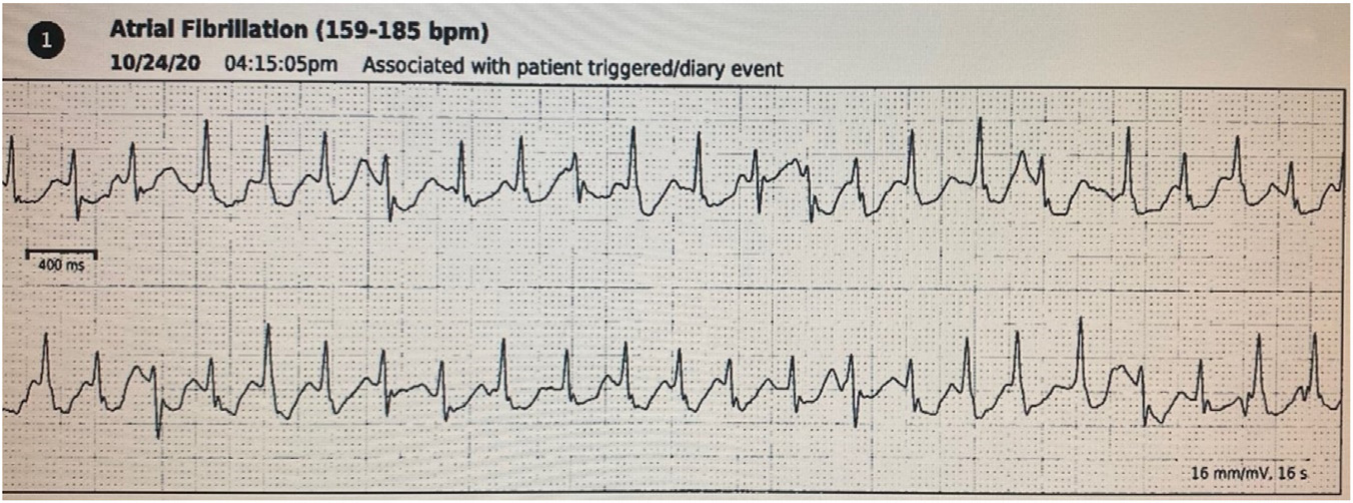
Rapid atrial fibrillation correlating with the patient’s symptoms.

Patient is a 79-year-old female who presented in clinic with a known history of symptomatic paroxysmal atrial fibrillation. Her symptoms consisted of fatigue, breathlessness, and profound palpitations. Outpatient monitoring (Figure 1) consistently revealed rapidly conducted atrial fibrillation, despite aggressive AV nodal–suppressing agents and antiarrhythmic agents including amiodarone. Both primary catheter ablation and AV junction ablation with pacemaker placement were discussed with the patient and her family. It was decided to proceed directly to AV junctional ablation and DDD pacemaker placement with HBP.

The procedure was performed using previously outlined techniques.^15^ After obtaining subclavian access, the Medtronic sheath was advanced into the right ventricle using a long guidewire. The guidewire and dilator were removed and the sheath was flushed. The bipolar His lead was advanced through the sheath. Alligator clips were placed on the distal poll of the lead and in the subcutaneous pocket. The tip of the lead was advanced slightly beyond the sheath, and mapping was performed to identify His deflection. A His deflection was identified. Unipolar pacing revealed appropriate His capture. The active fixation helix was then implanted into the septum at the site of the His deflection. Initially, a split His deflection was noted, followed by complete heart block. An atrial lead was positioned in the right atrial appendage using traditional techniques. The His lead was implanted in the CS port, the atrial lead was placed in the atrial port, and the previously placed RV lead was implanted in the RV port of the DDD pacemaker. The pocket was closed. Although we had planned to proceed with AV junction ablation, this was no longer necessary. Chest radiography was performed demonstrating lead placement in the right atrium, His bundle, and RV. The patient developed transient recovery of the His conduction; however, by the following day, the patient was in complete heart block again.

**Figure 2:**
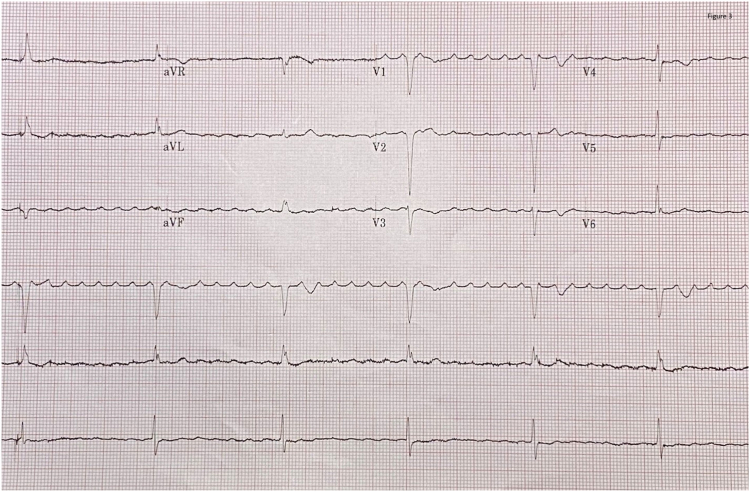
Electrocardiography demonstrating complete heart block with a Hisian escape rhythm.

**Figure 3:**
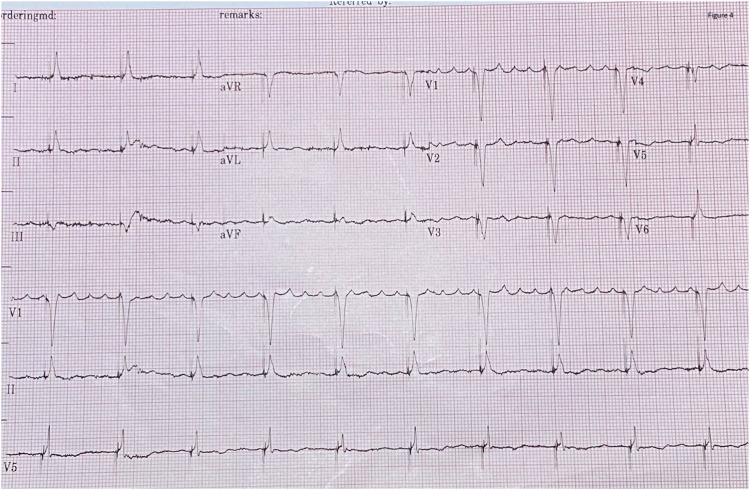
Electrocardiography demonstrating His bundle pacing.

At 18 months following the procedure, 12-lead electrocardiography was performed with her device inhibited (Figure 2), revealing complete heart block with a regular Hisian escape rhythm. Subsequent electrocardiography (Figure 3) demonstrated His bundle pacing.

## Discussion

This report describes a case of complete heart block due to His bundle injury via His lead placement. To our knowledge, this represents the first reported case of this complication. Given the variable nature of the His bundle location and depth in the septal wall, it may be impossible to fully ensure prevention of this complication. However, the use of more sophisticated mapping techniques could allow physicians to more carefully implant the His lead. New electroanatomic mapping systems such as EnSite NavX (Abbott Vascular), CARTO-3 (Biosense Webster), and Kodex-EPD (Philips Healthcare) all have potential to more precisely evaluate the position of the His bundle.^16^, ^17^, ^18^ More accurately analyzing the precise location of the His bundle could reduce the rates of this complication. Research now suggests that the depth of this His bundle is variable; however, more research into optimal depths for lead placement could prove fruitful.^15^

In practical application, knowledge about the possibility of complete heart block while performing HBP is an important factor to consider when examining forms of conduction system pacing. In this case, where an AV junction ablation was also planned, the transection of the His bundle had no effect on the procedural outcome. What compounds the issue of unexpected complete heart block due to HBP is the reduced reliability of HIS leads. As discussed previously, the rate of lead failure among HBP is approximately 5% to 6.7%.^13^ This could potentially create scenarios in which patients reliant on HBP due to iatrogenic complete heart block have lead failure.

From the perspective of the patient, it is important to include complete heart block when discussing informed consent. More research is needed to give an accurate estimate of the frequency of complete heart block following HBP, but ideally this information should also be made clear prior to the procedure. With the newfound popularity LBBP, or what is now considered conduction system pacing, the long-term risk of progressive conduction system block will likely decrease.

## Conclusion

Iatrogenic complete heart block should now be considered a possible complication of His bundle lead placement. More research should be done to fully elucidate its prevalence and what steps could be taken for prevention.

## Disclosures

The authors have no conflicts to disclose.
